# Congenital hepatic fibrosis with negative endoscopic evaluation of esophageal and gastric varices: A case report

**DOI:** 10.1097/MD.0000000000038424

**Published:** 2024-06-07

**Authors:** Lin Pan, Huaguo Shao

**Affiliations:** a Department of Ultrasound, Hangzhou Xixi Hospital, Hangzhou Xixi Hospital Affiliated to Zhejiang Chinese Medical University, Hangzhou, Zhejiang, China; b Institute of Hepatology and Epidemiology, Hangzhou Xixi Hospital, Hangzhou Xixi Hospital Affiliated to Zhejiang Chinese Medical University, Hangzhou, Zhejiang, China.

**Keywords:** congenital hepatic fibrosis, esophageal gastric varices, liver cirrhosis, portal hypertension

## Abstract

**Rational:**

Congenital hepatic fibrosis (CHF) is a rare autosomal recessive genetic disease, which is often diagnosed in children and young adults. The clinical manifestations of CHF were lack of specificity, mainly including portal hypertension related symptoms and signs, and normal or mildly abnormal liver function. When no obvious varices are indicated under endoscope, it can easily lead to misdiagnosis or missed diagnosis. We report this case in the hope of raising awareness of this disease.

**Patient concerns:**

A 31 years old male patient with major clinical manifestations of unexplained thrombocytopenia for 5 years.

**Diagnoses:**

Results of ultrasound, magnetic resonance imaging (MRI) and computed tomography portal venography (CTV) showed that patient had liver cirrhosis with portal hypertension and liver biopsy revealed CHF.

**Intervention:**

Patient received ursodeoxycholic acid tablets, fuzheng huayu capsule, ganshuang granule, etc for liver protection treatment.

**Outcomes:**

The condition of patient stabilized after symptomatic treatment. Spleen resection will be considered during follow-up.

**Lessons:**

This case reminds us that in case of patients with negative endoscopic evaluation, ultrasonic, computed tomography (CT) and MRI examination should be performed at the same time to determine whether patients have portal hypertension. When patients with normal or mildly abnormal liver function had unexplained liver cirrhosis complicated with portal hypertension, the possibility of CHF should be considered.

## 1. Introduction

Congenital hepatic fibrosis (CHF) is an autosomal recessive genetic disease first named by Kerr et al.^[1]^ CHF has many clinical manifestations including portal hypertension and its complications, normal or mildly abnormal liver function, kidney disease and no specific clinical symptoms.^[2]^ Due to the low incidence and limited reported cases of CHF, clinicians may have insufficient awareness and experience, which leads to misdiagnosis or missed diagnosis. Here we report a patient with CHF, who showed no obvious varices under endoscope, but diagnosed with cirrhosis complicated with portal hypertension and splenomegaly by ultrasound, computed tomography (CT) and magnetic resonance imaging (MRI) which showed obvious esophageal and gastric varices and patent umbilical vein. However, the patient did not exhibit cystic dilation of the intrahepatic bile ducts, and abnormalities of both kidneys, this case does not fit the criteria for Caroli syndrome.^[3]^ Combined with the laboratory examination data, clinical manifestations and the pathological results of ultrasound guided liver tissue biopsy, the patient was diagnosed with CHF. We collated the clinical data and discussed this case to improve the understanding of CHF.

## 2. Case presentation

A 31-year-old male patient was found to have a decrease in platelets to about 5 × 10^10^/L without no obvious incentive in the local hospital 5 years ago. The patient had no abdominal pain, distension, nausea, vomiting, chest tightness, shortness of breath or fever and showed no apparent signs of liver cirrhosis. From July 10 to July, 2021, the patient was hospitalized because of fever without an obvious trigger, while the platelet count was found to be 3.1 × 10^10^/L. Autoantibody test, hepatitis B antibody test and hepatitis C antibody test were negative. Abdominal enhanced CT suggested liver cirrhosis, splenomegaly, portal hypertension, esophageal and gastric varices, and splenic hilum varices. Symptomatic treatment led to a slight increase in platelets. For further treatment, the patient was admitted to our hospital on July, 2021.

When admitted to the hospital, the patient was conscious, with no jaundice in the skin or sclera, no liver palms or spider nevi. Cardiovascular examination revealed a regular heart rhythm, clear breath sounds were in both lungs. The abdomen was soft with no tenderness or rebound tenderness. The liver was not palpable below the ribs, and there was no percussion tenderness in the liver area. The spleen was palpable 5 cm below the ribs, with a soft texture and no tenderness. Murphy sign was negative, and there was no shifting dullness. No edema was present in the lower extremities. The pulse was 103 beats/min, the respiratory rate was 17 breaths/min, the blood pressure was 139/83 mm Hg, and the body temperature was 36.9°C. The patient father was healthy, mother had hypertension, 1 elder brother deceased due to enlarged liver and spleen with an unknown cause, and 2 elder sisters were healthy. There was no known hereditary, familial, infectious disease history or history of tumors. Laboratory examinations showed that white blood cell count was 2.9 × 10^9^/L, platelet count was 4.6 × 10^10^/L, and blood ammonia level was 39 μmol/L, total bilirubin was 20.65 μmol/L, direct bilirubin was 8.97 μmol/L, and prothrombin time coagulation time was 14.4 seconds. Fecal occult blood test was positive. Levels of albumin, globulin, cholinesterase, alanine aminotransferase, aspartate aminotransferase, tumor markers were normal. Hepatitis B surface antigen, hepatitis C antibodies, series of autoantibodies and autoimmune hepatitis antibodies were negative.

**Figure 1.**
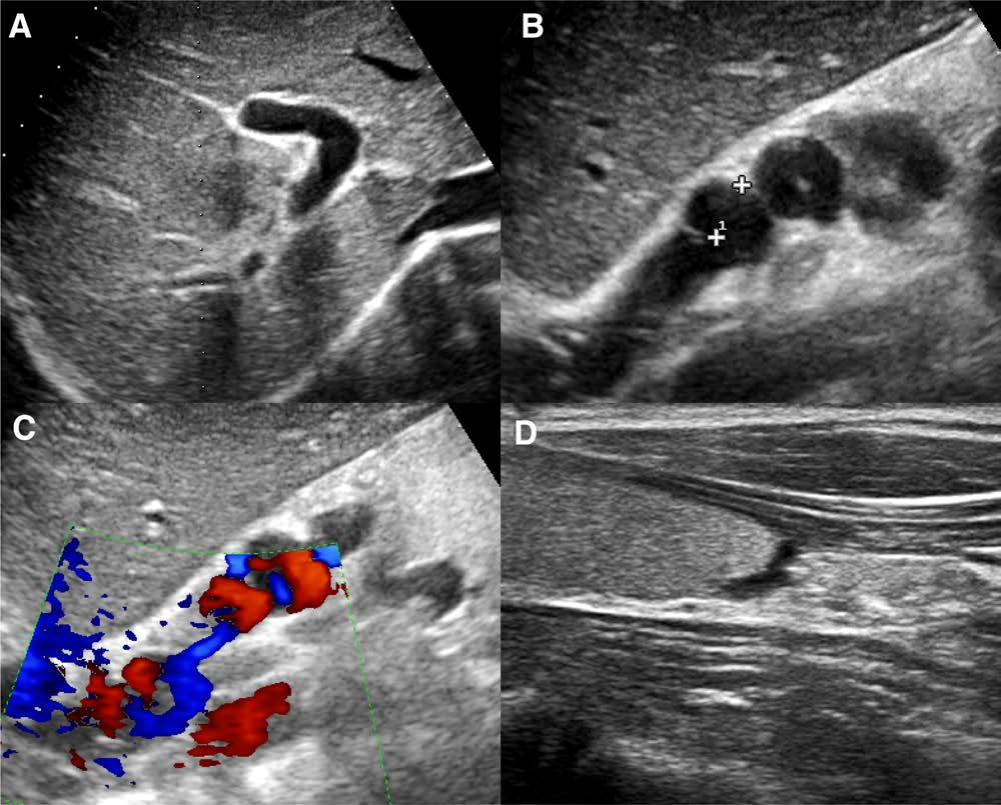
Ultrasound images. (A) Routine ultrasound showed hyperechoic and heterogeneous echogenicity of the liver, with no apparent dilation of the bile ducts. (B) Gastric filling ultrasonography showed a twisted and dilated tubular structure in the lower esophagus to the gastric fundus. (C) Color Doppler Flow Imaging of gastric filling ultrasonography showed twisted and dilated tubular structure filled with blood flow signals. (D) High-frequency probe indicated no apparent esophageal varices behind the thyroid in the cervical segment.

**Figure 2.**
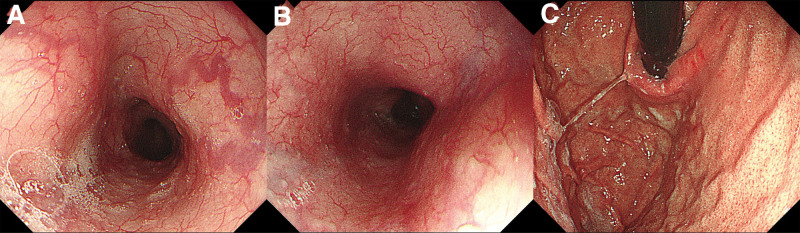
The mucosa of the (A) lower esophagus, (B) esophago-gastric junction and (C) gastric fundus showed scattered congestion, with a regular pattern of folds, and no apparent esophageal varices were observed.

**Figure 3.**
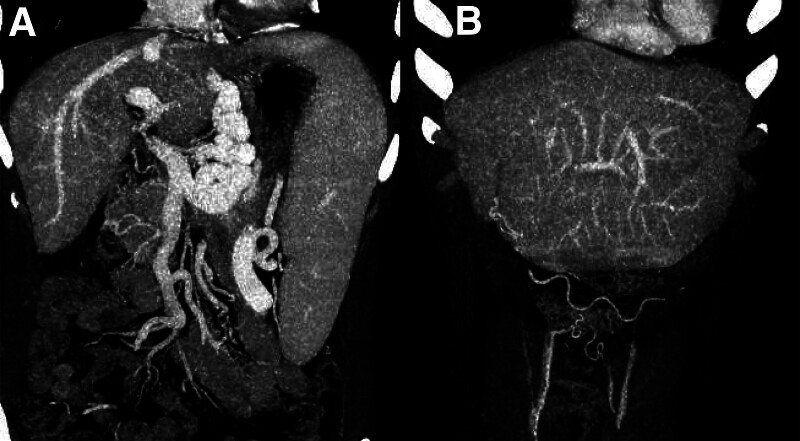
CTV Images. (A) Coronal view showed extensive tortuous dilated veins in the esophagus and gastric varices. (B) Coronal view showed recanalization of the umbilical vein extending to the umbilical region and superficial abdominal varices. CTV = computed tomography portal venography.

The initial ultrasound revealed liver cirrhosis with portal hypertension, splenomegaly, portal vein enlargement, and patent umbilical vein. Then, a gastric filling ultrasonography was done to assess the esophageal gastric varices. The results indicated that the esophageal gastric varices occurred in the lower part, with a width of approximately 1.1 cm, recommending endoscopic examination (Fig. 1). Endoscopic examination revealed reflux esophagitis, chronic superficial atrophic gastritis complicated with erosion and no apparent gastric varices (Fig. 2). Upper abdominal enhanced MRI showed liver cirrhosis, splenomegaly, abnormal collateral circulation, and slight dilation of intrahepatic and extrahepatic bile ducts. Computed tomography portal venography (CTV) showed multiple collateral circulations involving the abdominal wall veins, umbilical vein, esophagus, and gastric fundus (Fig. 3). Combining these findings, the patient was considered to have an external type of esophageal gastric varices, characterized by external and dilated mass of veins.

**Figure 4.**
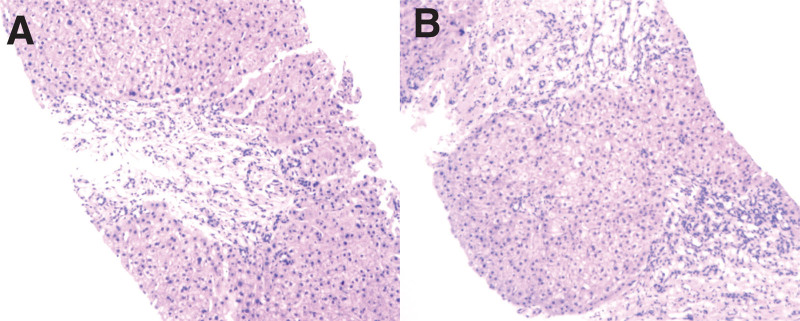
Pathology results. (A) showed slightly disordered lobule structure, significant proliferation of fibers in the portal area and increased bile duct proliferation (Hematoxylin and Eosin staining, ×200). (B) showed unclear normal liver lobule structure with the formation of pseudo-lobule and a large number of small bile duct fibrous strips separates the liver tissue (Hematoxylin and Eosin staining, ×200).

The patient had no history of liver-damaging drug intake, blood transfusion or unclean injections, liver diseases and heavy alcohol consumption, so, diagnoses of drug-induced hepatitis, viral hepatitis, alcoholic hepatitis, autoimmune hepatitis and metabolic hepatitis were excluded. Pathological findings indicated cirrhosis complicated with ductal plate malformation. The results of Immunohistochemistry and special staining were CD10 (+), CK7 (+), CK8/18 (+), HBcAg (−), HBsAg (−), PAS (+) (Fig. 4). According to medical history, imaging examinations, laboratory tests and liver biopsy pathology, the final diagnosis was CHF, portal hypertension, decompensated liver cirrhosis and splenic hyperfunction.

For treatment, medicines such as ursodeoxycholic acid tablets, fuzheng huayu capsule, ganshuang granule, etc were mainly based on liver protection because there was no specific treatment for CHF but only management complications. The patient was advised to rest in bed and consume a soft diet. After 15 days of hospitalization, the patient went well with no discomfort symptoms, was agreed to be discharged by doctor and recommended regular checkups every 3 months. The lifestyle recommendations for patient included rest, consuming a soft diet, maintaining regular bowel movements, avoiding fatigue, avoiding stimulating or spicy foods and absolute abstinence from alcohol. Three months later, the patient was hospitalized for follow-up. Laboratory and imaging examination showed no significant changes. However, the splenic hyperfunction persisted, and splenectomy surgery was recommended. The patient opted to delay the surgery and discharged after 5 days with liver protection medicine.

In subsequent follow-up, the patient continued to exhibit significant portal hypertension, splenic hyperfunction, and persistent low platelet level. To date, the patient does not receive further treatment.

## 3. Discussion

CHF is an autosomal recessive genetic disorder characterized by rare fibrous damage to the bile ducts, often occurring in children and adolescents. Studies found an association between CHF and mutations in the Polycystic Kidney and Hepatic Disease 1 gene (*PKHD1*), which encodes a transmembrane protein called fibrocystin.^[4]^ When fibrocystin expression is deficient, tissue-type plasminogen activator and plasminogen increase, causing degradation of β-chain fibrin and type IV collagen fibers and abnormity of cellular dedifferentiation and proliferation, leading to disruption or complete cessation of bile duct plate remodeling, which is referred to as bile duct plate malformation. Mutations in the *PKHD1* gene result in functional defects in fibrocystin in bile duct cells, leading to bile duct plate malformation, inflammatory necrosis of underdeveloped bile ducts, extensive collagen deposition, perivascular fibrosis of the portal vein and compression of the portal vein and its branches, ultimately progressing to a series of related clinical symptoms.

This case presented with unexplained thrombocytopenia, and the local hospital diagnosed cirrhosis without identifying a specific cause. Common causes of cirrhosis include:

- Viral hepatitis-related cirrhosis: The hepatitis B surface antigen and hepatitis C antibodies test of the patient were negative upon admission and the patient had no history of blood transfusions, unclean injections, or family history of liver disease. This cause was not considered.
- Drug-induced liver injury: The patient had no history of taking specific hepatotoxic drugs. This cause was not considered.
- Alcoholic cirrhosis: The patient had no history of long-term heavy alcohol consumption. This cause was not considered.
- Nonalcoholic fatty liver disease related cirrhosis: The patient had a normal body mass index (BMI), normal blood cholesterol and triglyceride levels, and imaging studies did not suggest fatty liver. This cause was not considered.
- Schistosome cirrhosis: The patient had no history of residing in endemic areas or schistosomiasis infection. This cause was not considered.
- Autoimmune liver disease: The test of autoimmune liver disease series and autoantibody series were negative. Indexex of liver function, including albumin, globulin, cholinesterase, alanine aminotransferase and aspartate aminotransferase, were normal. This cause was not considered.
- Congestive cirrhosis: The patient had no history of heart-related issues, and cardiac ultrasound shows normal results. This cause was not considered.
- Genetic metabolic liver diseases: The iron and copper metabolism were normal upon admission. Ophthalmic examination showed no evidence of Kayser-Fleischer rings. Hemochromatosis and Wilson disease were not considered.^[5]^

CHF is classified into 4 types: portal hypertension type, bile duct inflammation type, mixed type (portal hypertension combined with bile duct inflammation), and latent type.^[6]^ In China, the portal hypertension type is more common, clinically presenting with features such as portal hypertension and upper gastrointestinal varices and bleeding. Due to splenic hyperfunction, there may be a decrease in red blood cell, white blood cell, and platelet levels. Anemia may occur because of occult esophageal varices bleeding. Other manifestations include positive ascites signs and right upper abdominal pain. This particular case is characterized as portal hypertension type. The albumin, globulin, and cholinesterase levels of this patient were within normal ranges, indicated that the synthesis and reserve functions of the liver were not impaired. Ultrasound and enhanced MRI indicated no significant renal abnormalities, and renal function tests were basically normal. The reason why the initial symptom of this patient was thrombocytopenia, rather than the more common gastrointestinal bleeding was the patient had external gastric varices, resulting in negative endoscopic evaluation of esophageal and gastric varices, reducing the risk of gastrointestinal bleeding. The diagnosis of portal hypertension was confirmed by ultrasound, MRI, and CTV, preventing clinical oversight of gastrointestinal bleeding of the patient. The patient underwent gastric filling ultrasound examination with an empty stomach after orally ingesting 500 ml of contrast agent. The contrast agent is composed of powdered particles matured from corn, soybeans, etc. After gastric filling, the contrast agent appears as homogeneous hyperechoic echogenicity under ultrasound. Using a low-frequency ultrasound probe in conjunction with the patient supine, left lateral, right lateral, and sitting positions, various sections of the esophagus and stomach can be scanned, which is helpful for the detection of esophageal gastric varices.

CHF is a hereditary disease caused by bile duct plate malformation, leading to obstruction in the remodeling of dilated small tubes, making it difficult to form subsequent branches smoothly, forming the cystic cavities by the expansion of the confluence area.^[7]^ CHF often presents with cirrhosis as the initial symptom, and there are no specific imaging findings. For young patients with no obvious liver damage, unclear causes of cirrhosis, or portal hypertension, the possibility of CHF is often considered first.^[8]^

CHF often found in children, but in this case, it was a 31-year-old adult. The main reason was that adults usually had atypical and mild symptom.^[9]^ Children with CHF always characterized by severe complications of portal hypertension, while adults were asymptomatic. Just like this patient, before coming to our hospital, there were no abnormal results of blood tests, imaging and ultrasound examinations, resulting missed diagnosis.

The limitation of this case is that the patient did not undergo genetic testing. Currently, there is no effective treatment for inhibiting or reversing liver fibrosis in CHF patients. Therapy methods now primarily focuses on managing complications, such as splenectomy, trans-jugular intrahepatic portosystemic stent shunt, endoscopic sclerotherapy for esophageal and gastric varices, and liver transplantation. Liver transplantation can reduce mortality and is considered the optimal treatment for CHF at present.^[10]^

## 4. Conclusion

To sum up, the diagnosis of esophageal gastric varices is generally based on endo-scope evaluation, but it easily shows false negative when the varices appear to be ex-ternal. Clinically, CHF should be considered in cases of portal hypertension, normal or mildly abnormal liver function and cirrhosis with unknown causes. Liver biopsy is the gold standard for the diagnosis of CHF, which can be further supported by genetic testing. There is no radical cure for CHF, liver transplantation may be the best treatment currently.

## Acknowledgments

We would like to appreciate all patients who participated in this work.

## Author contributions

**Conceptualization:** Lin Pan, HuaGuo Shao.

**Investigation:** Lin Pan, HuaGuo Shao.

**Project administration:** HuaGuo Shao.

**Supervision:** HuaGuo Shao.

**Writing – original draft:** Lin Pan, HuaGuo Shao.

**Writing – review & editing:** HuaGuo Shao.
